# Effect of the English National Enhanced Service on weight management referral rate: an interrupted time-series analysis

**DOI:** 10.1136/bmjopen-2025-109943

**Published:** 2026-03-03

**Authors:** Stella J P Haffner, Richard John Stevens, Ben Amies-Cull, Laura Heath, Clare Bankhead, Paul Aveyard, Susan A Jebb

**Affiliations:** 1 Nuffield Department of Primary Care Health Sciences, University of Oxford, Oxford, UK; 2 Oxford and Thames Valley Applied Research Collaboration, University of Oxford, Oxford, UK; 3 NIHR Oxford Health Biomedical Research Centre, Oxford Health NHS Foundation Trust, Oxford, UK; 4 NIHR Oxford Biomedical Research Centre, Oxford University Hospital NHS Foundation Trust, Oxford, UK

**Keywords:** Obesity, Health policy, DIABETES & ENDOCRINOLOGY

## Abstract

**Objectives:**

To assess the impact of a National Enhanced Service (NES) incentive for weight management that financially rewarded practices for each eligible patient referred to a weight management programme.

**Design:**

Interrupted time-series analysis to examine the rate of weight management referral and weight management advice.

**Setting:**

Primary healthcare records from January 2018 to December 2024 in the Oxford Clinical Informatics Digital Hub, covering 8.3 million patients in 1198 primary care clinics around England.

**Interventions:**

NES payments to practices for weight management were introduced in April 2021.

**Results:**

The rate of referral increased from 1 referral per 1000 patients per month before the incentive to around 4 referrals per 1000 patients per month afterwards. There was no evidence that the increase differed by age, gender, ethnic group or socioeconomic status. The occurrence of weight management advice was unchanged by the introduction of the NES and was at least three times more common than referral to weight management services.

**Conclusions:**

The NES was associated with a fourfold increase in referrals to weight management services. However, clinicians are much more likely to offer advice rather than a referral to a weight management programme. There is a clear opportunity to improve outcomes for patients by encouraging greater use of referrals to effective weight management services in place of advice.

**STRENGTHS AND LIMITATIONS OF THIS STUDY**

- This analysis draws on data from 1198 different practices across England, providing a largely representative sample of the English patient population.
- This study’s parallel analysis of advice codes provides a helpful comparator by which to understand the changes in weight management referral codes.
- The current study uses electronic health record codes as a proxy for weight management referral. The increase in referral codes seen here may reflect a change in clinician coding behaviour rather than a change in referral patterns.
- The current analysis quantifies the rate of referral codes but cannot assess whether patients attended weight management services, as this information is not usually provided to general practitioners.

## Introduction

 Obesity is a global health concern.^1^ In England in 2022, 29% of adults were living with obesity, which is strongly linked to increased cardiovascular risks.^2^ The COVID-19 pandemic of 2020 onwards elevated concerns about health outcomes in this patient group who were more likely to be admitted to hospital, more likely to be admitted to intensive care, experienced longer hospital stays and had a higher mortality.^3 4^ During the COVID-19 pandemic, there was a rise in body weight^5^ and a sharp decline in referrals for weight management in the UK.^6^

Treating obesity routinely in primary care in England has been recommended by the National Institute for Health and Care Excellence since 2014, including opportunistic weight management advice and offers of referral to weight management services (WMS), regardless of whether weight is the presenting concern of the patient.^7^ Research has shown that this type of brief, opportunistic intervention is effective for weight loss, well-received by patients and acceptable to general practitioners (GPs).^8^ However, at the start of the COVID-19 pandemic, obesity was still rarely addressed with patients accessing primary care services.^8 9^

Financial incentives have been a main tool in health policy to change the behaviour of GPs. In 2004, the world’s largest pay-for-quality (P4Q) programme, the NHS Quality of Outcomes Framework (QOF), introduced indicators for preventative care to GP contracts to financially reward practices that achieve annual measures covering the management of conditions that tend to be common, chronic and of public health concern.^10^ However, weight management has not hitherto been a prominent target of these schemes. Apart from maintaining a register of adults living with obesity (indicator OB002 introduced in 2016), no other components of weight management were part of the contracts with English GPs prior to April 2021.^11^,^13^

As part of a national health reset prompted by the COVID-19 pandemic, the Government in England announced a National Enhanced Service (NES) incentive for weight management. The NES is an opt-in scheme for general practices in England that ‘aims to ensure that everyone living with obesity is offered support for weight loss’.^14^ It reimburses participating practices £11.50 for each patient identified as having obesity who is referred to a WMS. At the same time, the National Health Service (NHS) Digital Weight Management Programme (DWMP) was launched for people aged 18 and above living with obesity who also had hypertension and/or type 2 diabetes. While GPs could refer patients living with obesity to any available weight management service as part of the NES scheme, the DWMP ensured there was national availability of a suitable programme for people with increased cardiovascular risks. Early outcomes for the DWMP have been reported elsewhere.^15^

The aim of this study is to assess the effectiveness of the NES by quantifying the rate of WMS referrals made in primary care before and after the introduction of the NES.

## Methods

### Design

This study used electronic health records in the Oxford Clinical Informatics Digital Hub (ORCHID)^16^ to examine the rate of weight management referral and weight management advice provided in primary care clinics. This research was conducted as part of the MAINROUTE programme of work^17^ investigating the implications of the COVID-19 pandemic and measures taken in response to it and analysed in a series of interrupted time series (ITS) analyses. Relevant weight management advice and referral codes were identified through SNOMED-CT, the international standard of clinical vocabulary used in NHS electronic medical recording, to identify codes relevant to outcomes of interest.^18^

The data in ORCHID is anonymised and patients can opt out of data sharing. Data were analysed in R on secure ORCHID servers.

### Study population

We included all patients aged ≥18 who were contributing data to the ORCHID database during the study period (January 2018–December 2024).

### Exposures

The present study considered two exposures: the onset of the COVID-19 pandemic (which we approximated to March 2020, the first month of national lockdown) and the start of the NES incentive scheme (April 2021).

### Outcome measures

The aim of the NES was to increase the rate of referral to WMS for people living with obesity, with body mass index (BMI) thresholds for referral of 30 kg/m^2^ for people of white ethnicity or 27.5 kg/m^2^ for all other ethnic groups. The primary outcome was referral rate, calculated as the number of referral codes recorded by a practice divided by the number of active patients. We examined whether the impact of the incentive differed by gender, age, ethnicity and Index of Multiple Deprivation (IMD) quintile.

Since a discussion about weight could lead to advice from primary care staff to patients to lose weight, but not a referral, we also examined the rate of weight loss advice (calculated as the number of advice codes recorded by a practice divided by the number of active patients). There was no incentive payment for advice without referral to a weight management service.

### Analysis

We separately calculated rates of referral and rates of weight management advice per month in relation to the number of registered active patients and used segmented regression to test for differences in the rates before versus after the launch of the NES, controlling for calendar month to remove seasonal effects. This was implemented with a dummy variable for the pre- and post-COVID-19 pandemic period and for the pre- and post-policy period. All regression models included a random intercept term to account for variability between GP practices. We assumed no residual autocorrelation in our primary analysis and verified this assumption graphically (data not shown). In a post-hoc sensitivity analysis motivated by the lack of visible seasonal or monthly effects prior to the COVID-19 pandemic, we re-ran the analysis without adjusting for calendar month. In a further sensitivity analysis motivated by the reporting cycle used in the UK, we re-ran the analysis with an additional interaction between the start of the NES and the month of March, the last month in which P4Q measures can accumulate for the annual total return. Secondary outcomes were otherwise analysed with the same methods as the primary outcome. In the subgroup analyses, data are presented in the log scale to visualise comparisons between groups; the analysis itself was performed in the natural scale to emphasise the overall impact of the COVID-19 pandemic and the NES. We restricted the analysis to practices that were in the dataset for the entire study period. Data were extracted into Structured Query Language and analyses were carried out using R V.4.4.3 in RStudio^19^ with V.1.1-35.3 of the package lme4.^20^

Common covariates such as age, socioeconomic status and ethnicity are not time-varying or are very slow to change, so do not typically affect ITS^21^ and were therefore not controlled in this analysis. The covariate we were most concerned with is seasonality, since this has previously been shown to be linked to referral rates to the National Diabetes Prevention programme.^22^ We therefore controlled for calendar month and examined the residuals of our regression models for evidence of autocorrelation.

## Results

### Cohort description

A total of 8 256 249 patients 18 years old and above contributed to the data from 1198 practices. Further details about patient characteristics can be found in table 1 and, as expected, closely match the demographic profile of the adult population in England.

**Table 1:** Description of cohort demographic characteristics at the start of the study period (January 2018)

Patient characteristic	Total population (n=8 256 249)	
	N	%
Sex		
Female	4 119 111	49.9
Male	4 137 138	50.1
Age		
18–34	2 243 370	27.2
35–64	4 111 315	49.8
65+	1 901 564	23.0
Ethnicity		
Asian	702 006	8.5
Black	294 906	3.6
White	6 160 083	74.6
Ambiguous	571	0.00
Other	126 473	1.5
Mixed	107 008	1.30
Not specified	865 202	10.5
Index of Multiple Deprivation (IMD) quintile		
1 (most deprived)	1 529 235	18.5
2	1 677 829	20.3
3	1 640 519	19.9
4	1 665 795	20.2
5	1 718 548	20.8
Not specified	24 323	0.3

### Change in the rate of weight management referrals per month

**Figure 1:**
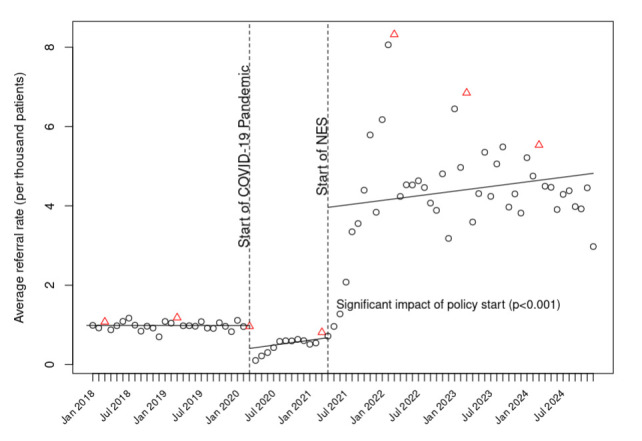
Weight management service referrals per month per 1000 registered patients before and after the introduction of National Enhanced Service (NES). End of financial reporting year (March) denoted by red triangles; all other months denoted by black circles. Solid lines denote fitted values after adjustment for month.

Prior to the COVID-19 pandemic, GPs coded approximately 1 referral per 1000 registered adult patients per month, with little change over time (figure 1 and online supplemental figure 1). After the March 2020 COVID-19 pandemic lockdown, there was an immediate drop in coded referrals, followed by a month-on-month gradual recovery. After April 2021, when the NES incentive was introduced, coded referrals rose rapidly to approximately 4 referrals per 1000 patients per month and remained at this level or above throughout the remainder of the analysis period.

In statistical modelling, both the COVID-19 pandemic (p=0.027) and the introduction of the NES (p<0.001) were associated with significant changes in coded referral rate (online supplemental table 1, column 1). This remained true in the alternative model that did not adjust for calendar month (online supplemental table 1, column 2) and in a log-scale model examining multiplicative effects (p<0.001, both for change at time of pandemic and for change at time of intervention).

### Differential changes in weight management referral rate by patient demographics

**Figure 2:**
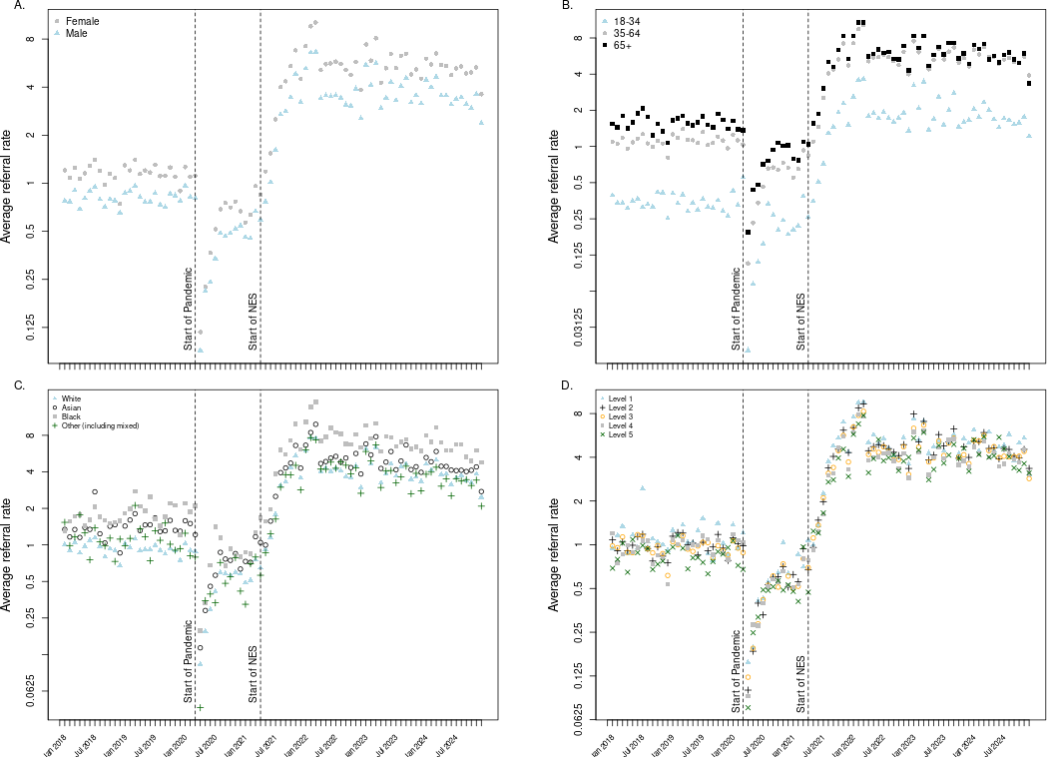
Comparison of weight management service referrals per month per 1000 registered patients across patient demographics. Average referral rate displayed in natural log scale to visualise multiplicative differences between demographic groups, instead of additive differences, across time series. (A) Sex. (B) Age. (C) Ethnic group. (D) Index of Multiple Deprivation quintile (1=most deprived). NES, National Enhanced Service.

Use of referral codes was more frequent in women than in men, and this remained true during the COVID-19 pandemic and after the introduction of the NES incentive (figure 2, panel a). Coded referrals were less frequent in younger adults than older adults, and again this remained true over time (figure 2, panel b). Coded referrals by ethnic group and by quintile of deprivation index are shown in figure 2 panels c and d, respectively, where there is no evidence that group trajectories changed substantively after the introduction of the NES.

### Change in weight management advice rate

**Figure 3:**
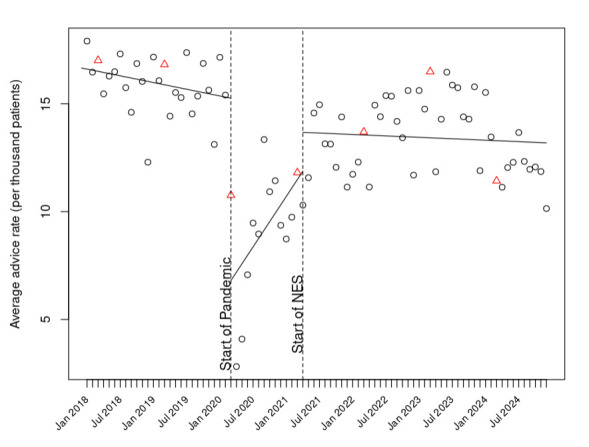
Instances of weight management advice per month per 1000 registered patients before and after the introduction of National Enhanced Service (NES). End of financial reporting year (March) denoted by red triangles; all other months denoted by black circles. Solid lines denote fitted values after adjustment for month.

Prior to the COVID-19 pandemic, approximately 15 instances of weight management advice were recorded per 1000 registered adult patients in a month, with a moderate downward trend (figure 3). After the March 2020 COVID-19 pandemic lockdown, there was an immediate drop in the coded provision of advice, followed by a gradual recovery. From April 2021, when the NES incentive was introduced, the rate of advice codes returned to approximately its pre-COVID-19 pandemic level, around 14 times per 1000 patients per month, and this remained consistent thereafter.

In statistical modelling, both the COVID-19 pandemic (p<0.001) and the introduction of the NES (p<0.001) were associated with significant changes in the rate of coded advice from the previous period, with a marked decrease at the onset of the COVID-19 pandemic, followed by gradual recovery. The trend after the introduction of the NES was similar to that observed pre-COVID-19 pandemic, with no evidence that the NES prompted an increase in the use of advice codes.

## Discussion

The introduction of the NES financial incentive was associated with a fourfold increase in the number of coded referrals for weight management. Referral codes occurred more frequently in women than men and in patients of a black ethnic background, and less frequently in younger adult patients. Baseline differences in referral rate appeared to be maintained across the time series, with no evidence that the NES had a differential effect on various demographic groups. There was no evidence that the policy change for referrals increased the use of weight management advice codes over and above pre-COVID-19 pandemic rates.

This analysis draws on data from 1198 different practices across England, with patients from varying ethnic backgrounds, geographic regions and levels of deprivation—providing a largely representative sample of the English patient population. Access to the number of active patients in a practice at any given month allowed this analysis to quantify the magnitude of change in the use of codes signifying referral to a weight management service, which provides a more complete picture of the effect of the NES compared with an analysis looking only at the absolute change in referrals. The findings from the primary analysis were stable across all versions of the model, supporting the robustness of this conclusion. A further strength of the present analysis is the parallel analysis of advice code rates. While the advice code data should be treated with caution, as it is a heterogeneous category including examples as broad as ‘recommended change in diet’ to examples as specific as ‘recommended change in sandwich intake’, it provides a helpful comparator which demonstrates consistent use over time in sharp contrast to the upturn in the use of the referral codes that were the specific target of the policy.

This analysis is subject to the usual limitations of routine health data, such as aggregate rather than individual-level information on socioeconomic status. In addition, the policy was introduced towards the end of the pandemic and we cannot separate the two. However, the step change supports the hypothesis that the changes observed are primarily attributable to the incentive, rather than a gradual trend, which would be more consistent with the phased ending of the COVID-19 pandemic. The aim of the policy was to change the uptake of behavioural support for weight loss. What increased was the use of qualifying codes, but this increase may have occurred because practitioners previously referred without coding it or used other codes that did not attract the fee and simply switched their coding behaviour. However, our use of all plausible codes that reflected weight management referral suggests that the increase in codes is real and not simply switching from one code to another. Incentives of various kinds are used to shape the behaviour of doctors in various health systems. This analysis primarily shows the power of financial rewards to change referral practices. Ideally, we would calculate rates per 1000 people with obesity, but unfortunately, this was not possible in this electronic health record database. GPs do not frequently record patients’ BMI, many patients lack a BMI record, and these two factors are likely to vary much more by practice than the true prevalence of obesity. Consequently, we calculated rates per 1000 adults who were registered for a whole calendar month. Similarly, the data does not identify which practices opted into the incentive. The model pre-specified in our analysis plan was consistent with our previous paper,^6^ but not necessarily optimal. Our sensitivity analyses explored some but not all potentially useful variations to the model (eg, generalised estimating equations). Given that the modelling details do not affect our graphs, that our p values for the intervention are conclusively significant or conclusively non-significant (p<0.001 in both primary and secondary analyses) and that our interpretation does not rest on the coefficients, such changes would not affect our conclusions. The primary limitation remains that this study design can only show association but not prove causality.

The criteria by which patients were eligible for referral were not the same for all patients; patients of a minority ethnic background were eligible for referral with a BMI of ≥27.5 kg/m^2^, while patients of a white ethnic background were eligible with a BMI of ≥30 kg/m^2^. This may explain why people from minority groups were more likely to be referred before and after the NES was introduced. This analysis was limited in its ability to describe the trajectory of referral rate following the introduction of the NES because there were only 44 months available after the policy came into practice, which precluded a multi-level model that integrated several different parameterisations of time. Several models were fit to understand the time-varying trends associated with the COVID-19 pandemic and the introduction of the NES; while the step change associated with the NES was present in all models, the estimate for the slope following the introduction of the NES changed depending on how the variable of calendar month was included in the model and depending on what month in the time series represented Time 0.

The introduction of the NES was followed by a marked change in GP behaviour with a fourfold increase in the use of referral codes for weight management. The national prevalence of adult obesity is 28% and we estimate that 48 patients per 1000 patients were referred every year and at least 150 per 1000 received advice. Taken together, this suggests that around 70% of eligible patients are offered some form of weight management intervention over the course of a year. However, advice codes occurred more than three times as frequently as referrals and may not have focused on weight management explicitly. Evidence from a trial comparing referral to a weight management service to advice alone found 2.4 kg greater weight loss after 1 year in the whole population.^8^ There is a clear opportunity to improve outcomes for patients by continuing to encourage greater use of referrals to WMS in place of advice. It is important to note that it is not clear from this analysis whether attendance at WMS increased, as GPs are usually not informed about attendance and do not code this. Recordings of weight management discussions taking place in routine care when the NES was operating suggest that the quality of discussion could be improved and simple training has been shown to improve the ability of staff in making referrals in a way that motivates attendance at a programme.^8 9^

The evidence on the effects of incentives on GP activity is complex and context-specific. An evaluation of the cost-effectiveness of the NES, together with referral to the DWMP, showed the programme was highly cost-effective, but that almost half of the total cost was attributable to the GP incentive associated with referral codes (B Amies-Cull *et al*, unpublished data). Although this present analysis shows that referrals are relatively stable, which could imply the effect of the incentive has waned and the service is now at a steady state, care needs to be taken in any change in approach. In Scotland, discontinuation of the QOF showed that removing incentives was associated with a decrease in associated clinical activities.^23^ Likewise, a recent systematic review reported that while recording of performance indicators consistently improved within the first year after the introduction of the respective incentive, gains in recording appeared to be lost after the incentive was removed.^24^

In summary, the NES was highly effective in significantly increasing rates of coded referrals to WMS from primary care for people living with obesity compared with pre-COVID-19 pandemic rates.

## Supplementary material

10.1136/bmjopen-2025-109943 online supplemental file 1

## Data Availability

The data that used in this study are not publicly available due to privacy, ethical, legal and information governance restrictions. Access to data for researchers who meet the criteria for access where costs of providing access to the data are covered are subject to mandatory, controlled review by the Primary Care Hosted Research Datasets Institutional Review Committee (PrimDISC) process (https://www.phc.ox.ac.uk/intranet/better-workplace-groups-committees-open-meetings/primdisc-committ…"

## References

[1] Obesity and overweight. https://www.who.int/news-room/fact-sheets/detail/obesity-and-overweight.

[2] NHS England Digital Health survey for England, 2022 part 2. https://digital.nhs.uk/data-and-information/publications/statistical/health-survey-for-england/2022-part-2.

[3] Gao M, Piernas C, Astbury NM (2021). Associations between body-mass index and COVID-19 severity in 6·9 million people in England: a prospective, community-based, cohort study. Lancet Diabetes Endocrinol.

[4] Altunkaya J, Piernas C, Pouwels KB (2024). Associations between BMI and hospital resource use in patients hospitalised for COVID-19 in England: a community-based cohort study. Lancet Diabetes Endocrinol.

[5] Valabhji J, Barron E, Bradley D (2021). Effect of the COVID-19 pandemic on body weight in people at high risk of type 2 diabetes referred to the English NHS Diabetes Prevention Programme. Lancet Diabetes Endocrinol.

[6] Heath L, Ordóñez-Mena JM, Aveyard P (2024). How has the COVID-19 pandemic affected the delivery of preventive healthcare? An interrupted time series analysis of adults in English primary care from 2018 to 2022. Prev Med.

[7] (2014). Weight management: lifestyle services for overweight or obese adults | guidance | NICE. https://www.nice.org.uk/guidance/PH53.

[8] Aveyard P, Lewis A, Tearne S (2016). Screening and brief intervention for obesity in primary care: a parallel, two-arm, randomised trial. The Lancet.

[9] Kebbe M, Jebb SA, Begh R (2022). General practitioner views on addressing weight opportunistically in primary care: An embedded sequential mixed-methods study. Patient Educ Couns.

[10] NHS England Digital Quality and outcomes framework (QOF). https://digital.nhs.uk/data-and-information/data-tools-and-services/data-services/general-practice-data-hub/quality-outcomes-framework-qof.

[11] The King’s Fund GP funding and contracts explained. https://www.kingsfund.org.uk/insight-and-analysis/long-reads/gp-funding-and-contracts-explained.

[12] NHS England Digital NHS payments to general practice - England, 2018/19. https://digital.nhs.uk/data-and-information/publications/statistical/nhs-payments-to-general-practice/england-2018-19.

[13] (2016). National institute for health and care excellence quality and outcomes framework (QOF) indicatory development programme. https://www.nice.org.uk/Media/Default/Standards-and-indicators/QOF%20Indicator%20Key%20documents/NM143-resource-impact.pdf.

[14] England, N. H. S. NHS England (2021). Enhanced service specification: weight management 2021/22. https://www.england.nhs.uk/publication/enhanced-service-specification-weight-management-2021-22/.

[15] Taylor K, Indulkar T, Thompson B (2024). Early outcomes of referrals to the English National Health Service Digital Weight Management Programme. *Obesity (Silver Spring*).

[16] de Lusignan S, Jones N, Dorward J (2020). The Oxford Royal College of General Practitioners Clinical Informatics Digital Hub: Protocol to Develop Extended COVID-19 Surveillance and Trial Platforms. JMIR Public Health Surveill.

[17] Nicholson BD, Ordóñez-Mena JM, Lay-Flurrie S (2022). Consultations for clinical features of possible cancer and associated urgent referrals before and during the COVID-19 pandemic: an observational cohort study from English primary care. Br J Cancer.

[18] NHS England Digital SNOMED CT. https://digital.nhs.uk/services/terminology-and-classifications/snomed-ct.

[19] R Core Team (2025). R: a language and environment for statistical computer. https://www.R-project.org/.

[20] Bates D, Mächler M, Bolker B (2015). Fitting Linear Mixed-Effects Models Using lme4. J Stat Softw.

[21] Szatkowski L, Aveyard P (2016). Provision of smoking cessation support in UK primary care: impact of the 2012 QOF revision. Br J Gen Pract.

[22] Koutoukidis DA, Barron E, Stevens R (2023). Association between the month of starting a weight management program and weight change in people at high risk of type 2 diabetes: A prospective cohort study. *Obesity (Silver Spring*).

[23] Morales DR, Minchin M, Kontopantelis E (2023). Estimated impact from the withdrawal of primary care financial incentives on selected indicators of quality of care in Scotland: controlled interrupted time series analysis. BMJ.

[24] Ho L, Mercer SW, Henderson D (2025). Effect of UK Quality and Outcomes Framework pay-for-performance programme on quality of primary care: systematic review with quantitative synthesis. BMJ.

