# Supplementary material for: Effect of the English National Enhanced Service on weight management referral rate: an interrupted time-series analysis
Source: BMJ Open. 2026 Mar 3;16(3):e109943. doi: 10.1136/bmjopen-2025-109943 (PMC12959051; doi:10.1136/bmjopen-2025-109943)
Supplement: online supplemental file 1 [file bmjopen-16-3-s001.docx]

SUPPLEMENT Figure 1


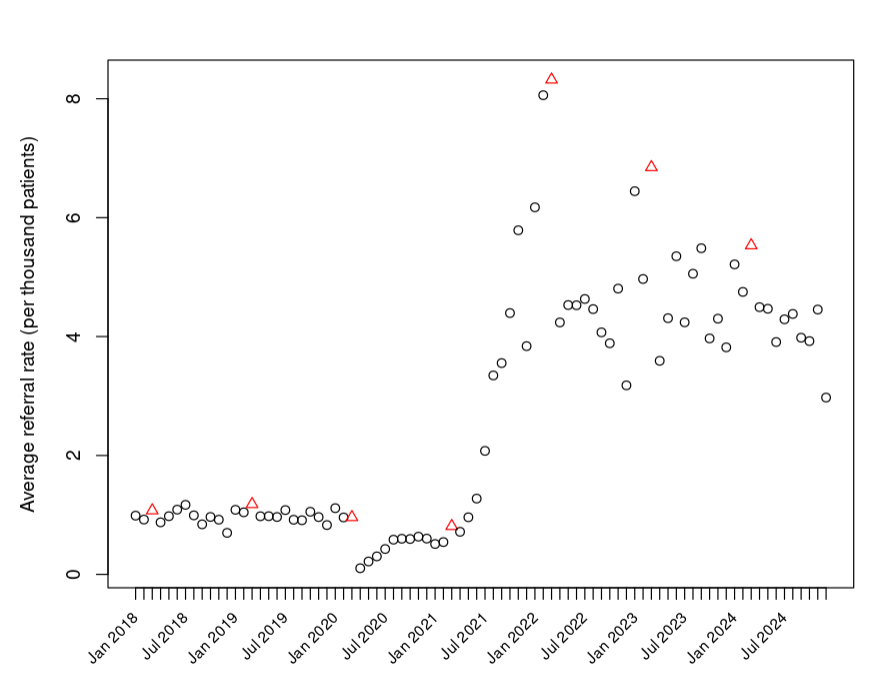


S1: *Weight management service referrals per month per 1,000 registered patients before and after the introduction of NES. End of financial reporting year (March) denoted by red triangles; all other months denoted by black circles.*

SUPPLEMENT Table 1

*Supplement Table 1.* Comparison of model estimates between a pre-specified model accounting for calendar month and a post-hoc model without calendar month as a covariate.

|  | **MODEL WITH CALENDAR MONTH** | | **MODEL WITHOUT CALENDAR MONTH** | |  |
| --- | --- | --- | --- | --- | --- |
|  | Coefficient* (95% CI) | | Coefficient* (95% CI) | |  |
| **Pre-COVID-19 pandemic** | | | | | |
| Intercept | 1.606 (1.236, 1.977) |  | 0.987 (0.695, 1,279) |  |  |
| Trend (per month) | 0.005 (-0.012, 0.022) |  | -0.001(-0.017, 0.0167) |  |  |
| **COVID-19 pandemic** | | | | | |
| Step change | -0.277 (-0.723, 0.168) | p < 0.0001 | -0.576 (-1.003, -0.150) | p = 0.02714 |  |
| Change in trend (per month) | -0.052 (-0.104, 0.000) |  | 0.021 (-0.030, 0.0716) |  |  |
| **Policy** | | | | | |
| Step change | 3.957 (3.515, 4.199) | p < 0.0001 | 3.286 (2.862, 3.709) | p < 0.0001 |  |
| Change in trend (per month) | 0.065 (0.014, 0.115) |  | -0.001 (-0.050, 0.047) |  |  |

* Referrals per thousand patients per month
