# Supplementary material for: Effect of the English National Enhanced Service on weight management referral rate: an interrupted time-series analysis
Source: BMJ Open. 2026 Mar 3;16(3):e109943. doi: 10.1136/bmjopen-2025-109943 (PMC12959051; doi:10.1136/bmjopen-2025-109943)
Supplement: Supplementary file 2 [file bmjopen-2025-109943.reviewer_comments.pdf]

PEER REVIEW HISTORY

BMJ Open publishes all reviews undertaken for accepted manuscripts. Reviewers are asked to complete a checklist review form and are provided with free text boxes to elaborate on their assessment. These free text comments are reproduced below.

ARTICLE DETAILS

Title (Provisional)

Effect of the English National Enhanced Service on weight management referral rate: an interrupted time series analysis

Authors

Haffner, Stella JP; Stevens, Richard John; Amies-Cull, Ben; Heath, Laura; Bankhead, Clare; Aveyard, Paul; Jebb, Susan A

VERSION 1 - REVIEW

Reviewer 1

Name Wollschlaeger, Daniel

Affiliation Institute for Medical Biostatistics, Epidemiology and Infomatics, University Medical Center, Johannes Gutenberg University Mainz, Biostatistics

Date 25-Sep-2025

COI None

Note: This review focuses on methods and statistics.

Haffner and co-authors present results from a study investigating the monthly rate of weight-loss management referral codes in UK GP practices in response to the COVID-19 pandemic and in introduction of the NES incentive system. Results from the iterrupted time series analysis indicate changes in code utilization associated with these two events.

Study design, data, and methods are in principle appropriate to address the research question. However, a number of major and minor methodological issues need to be addressed before publication can be considered.

Major issues

Since the analysis models a rate as the outcome, Poisson regression (or variants thereof accounting for possible overdispersion) would have been the natural choice for a statistical model with log eligible patient-time per GP per month serving as the offset. The qualitative pattern of results would likely be the same. Still, the authors should either change their

approach or argue convincingly why additive effects instead of multiplicative effects are to be expected.

A related issue is that linear regression assumes constant conditional error variance. Obviously, this is not the case for the data at hand, especially after the start of NES. Therefore, this needs to be taken into account - maybe even by directly modeling the variance in a GAMLSS model.

The statistical analysis seems to ignore the nested structure of the data because referral rates from the same GP are likely more similar to each other than rates from different GPs. This situation requires an appropriate mixed ((generalized) linear mixed model) or marginal (GLS, GEE) model. While the qualitative pattern of results will not be affected, error estimates and thus p-values will change.

A related issue is the autocorrelation structure of the data. This is not addressed.

The assumption of a linear secular trend in each time period is quite strong. Especially post NES start, the pattern looks quite nonlinear. (Penalized) regression splines or similar would be more flexible catching such patterns.

The authors calculate referral / advice code rates per 1000 active patients per month. However, they do not explain how the number of active patients is determined - is it the number of patients with at least one visit per quarter? Moreover, the denominator of this rate is - strictly speaking - incorrect. When the interest is in GP behavior, the number of referral codes should be relative to the eligible patient-time, only considering eligible patients with a BMI above 30 / 28.5. Any trend in the proportion of patients with high BMI among all patients would change referral numbers. Therefore, the post COVID-19 pandemic jump as well as the following trend could simply reflect changing obesity prevalence.

The authors chose to only include GP practices with data for the complete study period. This is potentially problematic as GP behavior from practices closing / opting out of NES during the study period and new GP practices or those newly opting in may be systematically different.

Related: What is the trend in the number of practices opting in to NES over time? I.e., is the increase in referral codes associated with individual GP behavior, or with more GPs opting in?

Minor issues

Even though the observed pattern of the averaged data follows a clearly visible pattern, it is good practice in ITSA analysis to add a plot without visual hints regarding the suspected breaks. This should be done here as well, possibly in the supplement.

When were semaglutide treatments introduced in the UK? The discussion could add a comment whether the number of prescriptions is high enough to represent a relevant alternative to weight management referrals.

The authors report that they considered socioeconomic status. However, they only used the Index of Multiple Deprivation (IMD) which is a region-based index. This is not the same as (individual) socioeconomic status.

The authors call age, SES, and ethnicity confounders. However, confounders need to be associated with the exposure as well as with the outcome. This is likely not the case here.

The authors report that they examined regression residuals. How and what for?

The start of NES more or less coincides with the end of the COVID-19 pandemic. This needs to be discussed.

In figure 1 and all corresponding figures, the x-axis should have calendar dates.

Throughout, including figures and tables, "pandemic" should be "COVID-19 pandemic" to make the article easier to understand in the future.

While not very ambiguous, the article should provide a rationale for choosing March 2023 as the start of the COVID-19 pandemic. Moreover, the article needs to discuss the end of this pandemic which is obviously less well defined, but still relevant with respect to GP practice attendance and GP behavior.

The authors twice state that the statistical analysis was carried out in RStudio. This is incorrect as RStudio is only a frontend / GUI for the statistical environment R. R should be properly cited. This applies to packages as well if these played a central role in the analysis.

The article deals with a situation that is very particular to the UK. It is not evident to what extent the research questions and results generalize beyond a regional interest.

p9 l151: "referral volume" -> "referral rate" (also p10 l167)

p12 l218: "has prompted a marked" - this is an observational study, residual confounding cannot be excluded, therefore causal interpretation is not adequate. Also p13 l241 "NES was highly effective in significantly increasing rates of coded referrals"

p12 l219: "frequency of referrals and advice codes suggest that most eligible patients are offered some form of weight management intervention" - how is that assessment possible without relating this to the obesity prevalence among patients?

Supplement Table 2. The caption is unclear. What exactly were the models that are being compared here?

Figure 1, 3: Caption should explain the lines from regression fits.

**Reviewer**	**2**
**Name**	**McLaughlin, Joanna**
**Affiliation**	**University of Bristol, Translational Health Sciences**
**Date**	**29-Sep-2025**

COI None

Thank you for the opportunity to review this manuscript. I find the work to be clearly presented and well-written. The methodology is sound and adequately documented. I have no comments to make on any elements which would in my opinion be necessary in terms of revision. I have a few comments which I offer as I feel they would add additional strength to the paper.

1. Line 220 states that most eligible patients are offered some form of intervention. If possible, please quantify this situation (both in terms of number of eligible patients in England, and in terms of the proportion of these receiving advice or referral) and comment on the target proportion of eligible patients to receive referral/remaining gap between eligibility and offer of support (particularly with regard to stated aims of the NES itself). doi: 10.1371/journal.pmed.1004282 may be an appropriate reference.

2. Please confirm that there were no other likely confounders in policy, incentives or practice which align with the timing of the NES introduction.

3. As these data have been examined by patterns of demographics in referral coding, examination by BMI category would hopefully be a straightforward addition and would offer insight in the discussion into the impact of the NES on reaching patients living with different levels of overweight and obesity.

4. Interpretation of the analysis of the advice codes would be improved by a statement on whether all patients with a referral code tended to also have advice codes or whether these are separate groups.

5. Any existing literature on the conversion rate of verbally delivered advice or completed referrals into coding for advice or referral would also aid interpretation.

6. Some discussion could be offered on the variation in availability or waiting times for weight management support (particularly local services and in person services rather than the national DWMP) and the role this may play in primary care professionals' motivation and willingness to refer.

VERSION 1 - AUTHOR RESPONSE

REVIEWER #1

1. Since the analysis models a rate as the outcome, Poisson regression (or variants thereof accounting for possible overdispersion) would have been the natural choice for a statistical model with log eligible patient-time per GP per month serving as the offset. The qualitative pattern of results would likely be the same. Still, the authors should either change their approach or argue convincingly why additive effects instead of multiplicative effects are to be expected.

The enhanced service incentive scheme was a response to the COVID-19 pandemic, as such this analysis follows from our previous analysis of the effect of COVID itself, and we followed the same

analysis strategy. Two valid issues are raised by the reviewer: modelling the outcome as continuous (vs. a Poisson model for counts or rates) and modelling effects as additive (rather than multiplicative, or additive on the log scale).

Firstly, we considered Poisson regression as an obvious choice for this data, but in this and other data sets we find the Normal approximation to the Poisson distribution is more robust against convergence problems. For example, in this data, a Poisson regression on counts per month, with random effects by practice, fails to converge with the following error message:

```
Warning messages:
1: In checkConv(attr(opt, “derivs”), opt$par, ctrl = control$checkConv,    :
Model failed to converge with max | grad | = 0.193189 (tol = 0.002, component 1)
2: In checkConv(attr(opt, “derivs”), opt$par, ctrl = control$checkConv,    :
Model is nearly unidentifiable: very large eigenvalue
- Rescale variables?; Model is nearly unidentifiable: large eigenvalue ratio
- Rescale variables?
```

We have consulted colleagues with expertise in mixed models and have taken the usual steps to resolve convergence problems by varying the optimisation algorithm, but the same applies with every algorithm available in the R package. The software does yield point estimates for fixed effects from the non-convergent model, and (to our surprise and scepticism) p-values, but not confidence intervals. With all algorithms, the point estimates and p-values from non-convergent models are consistent with the original findings presented: for the Reviewers’ information, we show a table below.

We agree with the reviewer’s hesitancy over this: because we are analysing data at the practice level, rather than aggregated nationally, the Normal approximation to the Poisson distribution will not be as good in the smaller practices. Nevertheless, given the strength of our p-value (especially for the primary analysis), we do not consider this to be a major threat to our conclusions.

Comparison of model estimates between a pre-specified model accounting for calendar month and a post-hoc model without calendar month as a covariate.

	**MODEL WITH CALENDAR MONTH**		**MODEL WITHOUT CALENDAR MONTH**		**NON-CONVERGENT POISSON MODEL**	
	Coefficient* (95% CI)		Coefficient* (95% CI)		Coefficient* (95% CI)	
**Pre-pandemic**						
Intercept	1.606 (1.236, 1.977)	--	0.987 (0.695, 1,279)	--	- 7.145 (na)	

Trend (per month)	0.005 (-0.012, 0.022)	--	-0.001(-0.017, 0.0167)	--	- 0.001 (na)	
**Pandemic**						
Step change	-0.277 (-0.723, 0.168)	p < 0.001	-0.576 (-1.003, -0.150)	p = 0.02714	-0.982 (na)	p < 0.001
Change in trend (per month)	-0.052 (-0.104, 0.000)		0.021 (-0.030, 0.0716)		0.060 (na)	
**Policy**						
Step change	3.957 (3.515, 4.199)	p < 0.001	3.286 (2.862, 3.709)	p < 0.001	3.286 (na)	p < 0.001
Change in trend (per month)	0.065 (0.014, 0.115)		-0.001 (-0.050, 0.047)		-0.001 (na)	

* Referrals per thousand patients per month. na = confidence intervals not able to be extracted due to non-convergent algorithm.

Secondly, even without a Poisson model, multiplicative effects could also be achieved in our linear model by log transformation of the outcome. In our previous analysis, we used an additive model which proved entirely adequate. (Heath et al., Preventive Medicine 181 (2024) 107923, https://doi.org/10.1016/j.ypmed.2024.107923.) Our statistical analysis plan for this manuscript therefore followed the same approach – we failed to anticipate how much better a multiplicative model would fit the effects, until we began work on the data. This gave us a dilemma: to proceed with our prespecified additive approach, or to make a *post hoc* change for better model fit at the expense of transparency and protocol-driven science. In the manuscript as submitted, we retained our protocol-specified analysis as the primary, but took a *post hoc* decision to use a logarithmic scale for the graphical subgroup analysis (Figure 2) to assist with interpretation.

In response to the Reviewer's remark, we have now repeated the primary analysis using a log transformation (an approximation is necessary for 0 observations – we replaced them with exp(-4)). In this model, the omnibus p-value for pandemic and the omnibus p-value for the policy are both <0.001. We have provisionally elected not to include this model in Table S1 because the coefficients have different units, representing a relative change per month instead of absolute change (referrals per thousand patients per month)

We hope the reviewer agrees with our interpretation, which is:

First, that in the light of the data obtained, the presence of a substantial change at the intervention time is not in doubt;

Second, that the major limitation is that a change at the intervention time does not prove causality (as in any interrupted time series study);

Third, that in the light of several uncertainties in the modelling decisions, numerical effect estimates should be interpreted cautiously and given less emphasis than the graphical presentation.

We hope that the reviewers and editors agree that the paper as written correctly reflects this interpretation.

Following the reviewer's comment, we have run a new model to assess the multiplicative effects discussed. We are hesitant to endorse this model because all model estimates were significant at a level of 2e-16, which may be due to the recoding of zeros after log scaling the data. In order to, run a model without observations with zero referrals, we tested an additional model that aggregated referral rate across all practices each month. Knowing that this model would be underpowered, we are not surprised that pandemic no longer showed a significant relationship with referral rate however there is still a significant relationship between the NES policy and referral rate.

2. A related issue is that linear regression assumes constant conditional error variance. Obviously, this is not the case for the data at hand, especially after the start of NES. Therefore, this needs to be taken into account - maybe even by directly modeling the variance in a GAMLSS model.

This is a good point, especially since practices vary in size, which will drive substantial heteroscedasticity. To examine the impact of this, we have now run an additional linear model containing data only from the medium sized GP practices. As with our original analysis, we have performed a joint p-value test to estimate the effect of the COVID-19 pandemic and the NES policy. This test shows a significant effect of the NES policy ($p < 0.001$) and a non-significant effect of the pandemic ($p = 0.220$). We think this confirms that our original finding is robust – that is, the "effect" of policy remains statistically significant even in a dataset that is underpowered to detect any effect of the COVID-19 pandemic.

3. The statistical analysis seems to ignore the nested structure of the data because referral rates from the same GP are likely more similar to each other than rates from different GPs. This situation requires an appropriate mixed ((generalized) linear mixed model) or marginal (GLS, GEE) model. While the qualitative pattern of results will not be affected, error estimates and thus p-values will change.

We agree that the nested structure is an important feature of this dataset, which is why the analysis presented in the manuscript is a mixed effects model, including a random intercept to address practice-level differences. This has now been highlighted in the methods section on line 113: "All regression models included a random intercept term to account for variability between GP practices." However, due to the constraints of electronic health records, there is no available data on the differences between clinicians themselves. Within the UK, it is commonplace for patients to attend the same GP practice but see different clinicians each time, mitigating potential clinician-level effects.

4. A related issue is the autocorrelation structure of the data. This is not addressed.

This was considered in the analysis plan, but it is not addressed in the manuscript – we thank the reviewer for bringing the oversight to our attention. We did consider whether to include an autocorrelation term in the model. Bernal et al. (International Journal of Epidemiology, 2017, Vol. 46, No. 1) note that "in epidemiological data ... residual autocorrelation is rarely a problem" provided that other factors – especially, autocorrelation – have been correctly adjusted for. This is supported by our own experience in CPRD data and, more comprehensively, the systematic review of Turner et al. (2021; BMC Med Res Methodol 21:134, Table 9). We therefore designed the analysis under the assumption that residual autocorrelation, after adjustment for seasonality (monthly effects as indicator variables), would be negligible. To confirm this, we produced an autocorrelation function plot of the residuals.

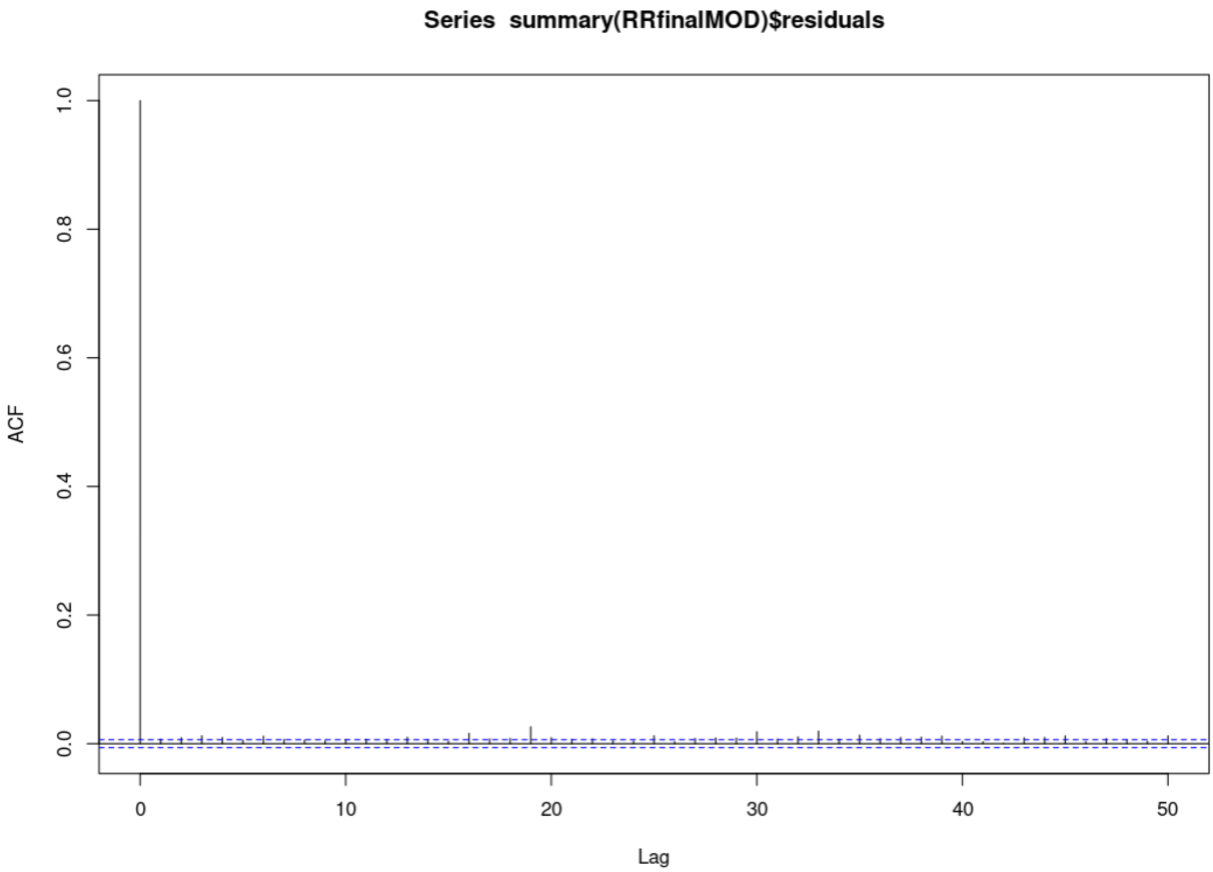

These exceptionally low autocorrelations will seem spurious to statisticians familiar with, for example, economic time series' data, but they are well within the plausible range reported in the Turner et al. review.

We have now added to the methods the following sentence:

We assumed no residual autocorrelation in our primary analysis, and verified this assumption graphically (data not shown).

We are satisfied with this approach for these results, whereas we might have been less satisfied if our primary p-value was less clear-cut (see also our reply to the Reviewer's first comment).

5. The assumption of a linear secular trend in each time period is quite strong. Especially post NES start, the pattern looks quite nonlinear. (Penalized) regression splines or similar would be more flexible catching such patterns.

Yes, the pattern looks less than linear. Some of that nonlinearity is accounted for by the monthly indicator variables but even so, linearity is at best a first-order approximation. We think there is not sufficient data (especially, within each time period) to fit splines while also modelling 'seasonality' (monthly effects). (In our experience, other approaches (e.g. sinusoidal) to seasonality do not fit well in health data.) It is a limitation that the data series is too short to support anything other than a first order linear approximation.

Given the strength of the nominal p-values and the effects visible in the graph, we think it unlikely that any alternative modelling of secular trends would yield different conclusions, but, again, we think it would be a mistake to over-emphasize the actual numerical effect estimates in the light of such modelling uncertainty. We think the statistical significance is robust enough and the graphical presentation is useful and have tried to avoid over-interpreting the numerical effect estimates in the discussion.

6. The authors calculate referral / advice code rates per 1000 active patients per month. However, they do not explain how the number of active patients is determined - is it the number of patients with at least one visit per quarter? Moreover, the denominator of this rate is - strictly speaking - incorrect. When the interest is in GP behavior, the number of referral codes should be relative to the eligible patient-time, only considering eligible patients with a BMI above 30 / 28.5. Any trend in the proportion of patients with high BMI among all patients would change referral numbers. Therefore, the post COVID-19 pandemic jump as well as the following trend could simply reflect changing obesity prevalence.

We considered that it was ideal to calculate rates per 1000 people with obesity, but unfortunately this was not possible. GPs do not frequently record patients' BMI, many patients lack a BMI record, and these two factors are likely to vary much more by practice than the true prevalence of obesity. Consequently, we calculated rates per 1000 adults who were registered for a whole calendar month. However, those who transfer out of a practice become inactive, as do patients who opt out of sharing their data with CPRD.

7. The authors chose to only include GP practices with data for the complete study period. This is potentially problematic as GP behavior from practices closing / opting out of NES during the study period and new GP practices or those newly opting in may be systematically different.

To be clear, practices that are not included for the whole study period are those that opted into or out of the sharing of their data with CPRD. We do not know which practice opted into the obesity NES, and that is not a criterion for inclusion in this study.

We agree that practices opting in and out of data collection could differ systematically from those that were included for the whole period. If we did not take this into account, this could induce

confounding with time – we would not be comparing like-with-like, as the population of practices changed over time. To prevent this, we restricted attention to practices that shared data for the entire study period.

In other words, we considered the issue the reviewer raises, and this is the very reason we made this choice.

8. Related: What is the trend in the number of practices opting in to NES over time? I.e., is the increase in referral codes associated with individual GP behavior, or with more GPs opting in?

It is true that changes in the trend of practices opting-in to the NES could be expected to change referral rates. However, because this was an extremely attractive policy for GP practices the rate of opt-in was 93% from the inception of the policy. We have interpreted the trend seen here to be a true increase in referral coding.

9. Even though the observed pattern of the averaged data follows a clearly visible pattern, it is good practice in ITSA analysis to add a plot without visual hints regarding the suspected breaks. This should be done here as well, possibly in the supplement.

Thank you for your comment. We have plotted a new graph without trend lines and time series breaks and included it in the supplementary materials.

10. When were semaglutide treatments introduced in the UK? The discussion could add a comment whether the number of prescriptions is high enough to represent a relevant alternative to weight management referrals.

We did not include prescription of weight loss drugs as part of this analysis as they were not eligible for the referral fee. Moreover GPs were not allowed to prescribe GLP-1 medications for obesity at the time of this analysis. Patients who require treatment for severe and complex obesity could be referred to clinics in secondary care (which would be reimbursed under the NES) and GLP-1 medications could be prescribed. However, supply problems meant that there was almost no prescription until the summer of 2024.

11. The authors report that they considered socioeconomic status. However, they only used the Index of Multiple Deprivation (IMD) which is a region-based index. This is not the same as (individual) socioeconomic status.

GPs do not record a patient’s socioeconomic status, so the only indicator open to us is the postcode in which they live. This leads to the IMD score of the lower super output area from the patient’s area of residence, which is local rather than regional. We think it is justified to write that there is no evidence of variation with socioeconomic status, albeit limited by the data available on this. This is one of the “usual limitations of routine health data” that we refer to on line 202 in the Discussion.

12. The authors call age, SES, and ethnicity confounders. However, confounders need to be associated with the exposure as well as with the outcome. This is likely not the case here.

The reviewer is correct. We have replaced ‘confounder’ with ‘covariate’.

13. The authors report that they examined regression residuals. How and what for?

This is stated in the context of adjustment for calendar month, and refers to the examination of residual autocorrelation: see item 4 above.

14. The start of NES more or less coincides with the end of the COVID-19 pandemic. This needs to be discussed.

The NES was introduced partly in response to the much higher morbidity and mortality from COVID of people with obesity. We think it is difficult to define the end of the pandemic, since partial lockdown measures of varying strictness (e.g. ‘rule of 6’) were in force until end of July 2021. We have added a comment to acknowledge that at least part of the step change could be due to the end of the pandemic. However, general practice had recovered to a normal state much earlier in the pandemic than this and we do not think the step change can be explained by this.

Line 206-209: *In addition, the policy was introduced towards the end of the pandemic and we cannot separate the two. However the step change supports the hypothesis that the changes observed are primarily attributable to the incentive, rather than a gradual trend which would be more consistent with the phased ending of the COVID-19 pandemic.*

15. In figure 1 and all corresponding figures, the x-axis should have calendar dates.

All x-axes have now been changed from time in months to calendar dates.

16. Throughout, including figures and tables, "pandemic" should be "COVID-19 pandemic" to make the article easier to understand in the future.

All mentions of the pandemic in the manuscript now describe it as the “COVID-19 pandemic”.

17. While not very ambiguous, the article should provide a rationale for choosing March 2023 as the start of the COVID-19 pandemic. Moreover, the article needs to discuss the end of this pandemic which is obviously less well defined, but still relevant with respect to GP practice attendance and GP behavior.

We chose the start as March 2020 because the nation went into lockdown in March 2020, and we now describe this in the Methods as

Line 94-95: *"the start onset of the COVID-19 pandemic (which we approximated by March 2020, the first month of national lockdown)"*

There was no clear end to the pandemic for general practice. Arguably, practice returned to more or less a normal pattern by early 2022.

18. The authors twice state that the statistical analysis was carried out in RStudio. This is incorrect as RStudio is only a frontend / GUI for the statistical environment R. R should be properly cited. This applies to packages as well if these played a central role in the analysis.

We have now made the appropriate edit on line 124

> *... analyses were carried out using R version 4.4.3 in RStudio[26] ~~version 4.4.3~~ with version 1.1-35.3 of the package lme4[27].*

19. The article deals with a situation that is very particular to the UK. It is not evident to what extent the research questions and results generalize beyond a regional interest.

We agree that examinations of the effect of policy necessarily examine the effect of a policy introduced in one country and in one health system. However, the generally relevant question here is whether it is useful to incentivise GPs to offer treatment for obesity – we show clearly that it is. Pay for performance systems are common policy tools used the world over.

20. p9 l151: "referral volume" -> "referral rate" (also p10 l167)

We have now made the appropriate edits.

21. p12 l218: "has prompted a marked" - this is an observational study, residual confounding cannot be excluded, therefore causal interpretation is not adequate. Also p13 l241 "NES was highly effective in significantly increasing rates of coded referrals"

We have changed "has prompted" to "was followed by".

22. p12 l219: "frequency of referrals and advice codes suggest that most eligible patients are offered some form of weight management intervention" - how is that assessment possible without relating this to the obesity prevalence among patients?

We related our calculated referral rate to a national prevalence of obesity of 28%. Specifically, 280 of every 1000 are eligible for referral to weight management services and can trigger the £11.50 NES payment. In the study period, approximately 48 patients per 1000 patients were referred every year and at least 150 per 1000 received advice. We estimated this to be more than 70% of patients with obesity who were offered some sort of weight management support.

We have added this additional detail to the paper:

Line 229-232: *"The national prevalence of adult obesity is 28% and we estimate that 48 patients per 1000 patients were referred every year and at least 150 per 1000 received advice. Taken together, this suggests around 70% of eligible patients are offered some form of weight management intervention over the course of a year."*

23. Supplement Table 2. The caption is unclear. What exactly were the models that are being compared here?

We have removed this Table from the Supplement, and removed from the text the reference to it.

In answer to the reviewer's query: each column of this table corresponded to one of the panels of Figure 2, showing the coefficients and p-values for the main effects (pandemic and policy) when these subgroups (sex, age, ethnic group, IMD quintile) are included as covariates. To overcome the difficulty with the logarithm of 0 (see response to Reviewer 1 item 1), in this graphical analysis we aggregated data across all centres – so that a referral account of 0 never occurred. We are satisfied with this as a way to conduct the graphical analysis, but not as a formal modelling analysis due to the loss of statistical power.

24. Figure 1, 3: Caption should explain the lines from regression fits.
We have added "Solid lines denote fitted values after adjustment for month." to each caption.

REVIEWER #2

1. Line 220 states that most eligible patients are offered some form of intervention. If possible, please quantify this situation (both in terms of number of eligible patients in England, and in terms of the proportion of these receiving advice or referral) and comment on the target proportion of eligible patients to receive referral/remaining gap between eligibility and offer of support (particularly with regard to stated aims of the NES itself). doi: 10.1371/journal.pmed.1004282 may be an appropriate reference.

See response to Reviewer 1 point 22.

2. Please confirm that there were no other likely confounders in policy, incentives or practice which align with the timing of the NES introduction.

Weight management policy is continually evolving. In 2019 the National Diabetes Prevention programme began a digital alternative to the face-to-face programme, in April 2020 the Digital Weight Management Programme commenced and in September 2020 the Pathway to Remission, a total diet replacement programme was introduced specifically for people with recently diagnosed with type 2 diabetes and obesity. These added to the range of programme types available but to our knowledge, capacity was not previously saturated and this would not be expected to directly impact the referral rate at exactly the time we see a step change at the same time as the introduction of the NES.

We have also discussed the availability of semaglutide treatment in our response to Comment #10 from Reviewer #1 and concluded that this was unlikely to impact the rate of referral coding.

3. As these data have been examined by patterns of demographics in referral coding, examination by BMI category would hopefully be a straightforward addition and would offer insight in the discussion into the impact of the NES on reaching patients living with different levels of overweight and obesity.

See response to Reviewer 1 point 6. The problem here is that BMI is rarely recorded by GPs, and many people lack a BMI in the GP record. As such, it is not possible to calculate reliable denominators for these rates.

4. Interpretation of the analysis of the advice codes would be improved by a statement on whether all patients with a referral code tended to also have advice codes or whether these are separate groups.

We agree with the reviewer that data on the co-incidence between advice and referral would give us a better understanding of weight management support in primary care. Unfortunately, due to the nature of the data presented here, it is not possible to analyse advice and referral codes at the patient-level.

5. Any existing literature on the conversion rate of verbally delivered advice or completed referrals into coding for advice or referral would also aid interpretation.

We are not aware of any literature that examines this but agree that it is an important factor in understanding the referral process, and we have sought to be clear in the paper that we can only use codes as an indicator of practice. We have acknowledged this as a limitation.

6. Some discussion could be offered on the variation in availability or waiting times for weight management support (particularly local services and in person services rather than the national DWMP) and the role this may play in primary care professionals' motivation and willingness to refer.

Based on the data presented here we are unable to speculate on how wait times may have impacted referral coding or willingness to refer. However, evidence from Tier 2 service providers (Fong et al., 2023: **doi.org/10.1111/cob.12629**) suggests that there is great variation both in service availability and capacity across the country for locally commissioned services.

VERSION 2 - REVIEW

Reviewer 1

Name **Wollschlaeger, Daniel**

Affiliation **Institute for Medical Biostatistics, Epidemiology and Infomatics, University Medical Center, Johannes Gutenberg University Mainz, Biostatistics**

Date **14-Dec-2025**

COI

I thank the authors for extensively responding to the issues raised in my previous review, and for taking many of these issues into account in the revised manuscript. While no major concerns remain, I still see room for improvement and thus recommend minor revisions before publication.

1) I agree that the pre-specified primary analysis using a linear model can remain as the main pattern of results is likely robust against again particular modeling choice. However, in particular regarding the sensitivity analysis using Poisson regression, an additional approach should be explored. However, as the authors confirm, there is substantial heteroscedasticity in the data. As such, it is advised to use an estimator for the covariance matrix of estimated coefficients that takes heteroscedasticity into account. A possible approach is offered by R package sandwich using a function such as vcovHC() together with coeftest() from package lmtest.

2) The convergence issues for the generalized linear mixed model (implemented using the lme4 package for R) reflect difficulties also reported by others. Even if results from a model with failed convergence may be printed out, these results should be ignored. Instead, repeating a suggestion from my earlier review, it is worth implementing a generalized estimating equations Poisson regression model (e.g., using the geepack package for R) - keeping in mind the correct choice for the offset. Coefficients from such a Poisson model with a log-link are easier to interpret than coefficients from a linear model for the log-rate. Furthermore, the Poisson model does not require replacing 0 observations with an arbitrary non-zero number.

3) I agree with the authors that given the adjustment for calendar month, autocorrelation may not represent a substantial issue, and thus see no further need to address this issue. Still, I would like to point out that a linear GLS model can handle AR1 errors, and would have been a viable choice since there is no interest in estimating the random effects variance anyway. Function gls() from R package nlme would offer a way to implement such a model.

4) In response to the original issue 6, the authors agree that ideally, rates should be based on the number of obese patients per GP per month, yet this information is not available. This limitation should be explicitly mentioned in the discussion.

5) In response to the original issue 7, the authors agree that GP practices that opted in to sharing of their data for the wholde time period may systematically differ from those

practices not analyzed. This limitation sould be explicitly discussed as it is a question of how representative the obtained data are.

6) In response to the original issue 7, the authors provide a reasonable explanation which is, however, speculative. As such, it should be mentioned in the limitations.

7) In response to the original issue 11, the authors provide a reasonable argument. The issue remains, however, that individual SES data was not available, and this should be mentioned in the limitations.

8) p10 l187 "There was no evidence that the policy changed increased " -> delete "increased"

**Reviewer**	**2**
**Name**	**McLaughlin, Joanna**
**Affiliation**	**University of Bristol, Translational Health Sciences**
**Date**	**15-Dec-2025**
**COI**	

I have reviewed the responses to the reviewers' comments and I am satisfied that the comments have all been satisfactorily addressed.

I note the addition at line 156 "and in a log-scale model examining multiplicative effects (Supplement Table 1, column 3)." but do not see that this column has been included. Please correct.

Also note typo line 190: 'changed' should be 'change'.

VERSION 2 - AUTHOR RESPONSE

REVIEWER #1

I thank the authors for extensively responding to the issues raised in my previous review, and for taking many of these issues into account in the revised manuscript. While no major concerns remain, I still see room for improvement and thus recommend minor revisions before publication.

1) I agree that the pre-specified primary analysis using a linear model can remain as the main pattern of results is likely robust against again particular modeling choice. However, in particular regarding the sensitivity analysis using Poisson regression, an additional approach should be explored. However, as the authors confirm, there is substantial heteroscedasticity in the data. As such, it is advised to use an estimator for the covariance matrix of estimated coefficients that takes heteroscedasticity into account. A possible approach is offered by R package sandwich using a function such as vcovHC() together with coeftest() from package lmtest.

Unfortunately, in our secure data environment, installation of new R packages is not within our control, but falls under a committee approvals process. Another consequence of our secure data environment is that the data cannot be accessed outside the UK - which applies to author SH, who conducted all the original analyses, but is now studying in Copenhagen and is no longer employed by us. With both of these constraints, we are not able to provide the analyses (GEEs, sandwich estimates, the reviewer suggests in the timeframe that the journal requests (or indeed, for many months).

Given that these modelling issues do not affect our graphs; that our p-values for the intervention are conclusively significant or conclusively non-significant (p<0.001 in both primary and secondary analyses); and that our interpretation does not rest on the coefficients - we hope the reviewer, and the editors, will agree that our conclusions are not threatened or undermined by the statistical modelling challenges.

2) The convergence issues for the generalized linear mixed model (implemented using the lme4 package for R) reflect difficulties also reported by others. Even if results from a model with failed convergence may be printed out, these results should be ignored. Instead, repeating a suggestion from my earlier review, it is worth implementing a generalized estimating equations Poisson regression model (e.g., using the geepack package for R) - keeping in mind the correct choice for the offset. Coefficients from such a Poisson model with a log-link are easier to interpret than coefficients from a linear model for the log-rate. Furthermore, the Poisson model does not require replacing 0 observations with an arbitrary non-zero number.

We note and appreciate the Reviewer's expertise in this area of statistics. We apologise for not recognising GEEs sooner as a solution to the convergence problems, but as noted above, the necessary packages are not currently available in our secure data environment. Again, if the results were less conclusive we would request a (long) extension from the journal and apply for the installation of new packages, but we think – and we hope the reviewer and editors agree – that there is not sufficient doubt to justify such a delay.

3) I agree with the authors that given the adjustment for calendar month, autocorrelation may not represent a substantial issue, and thus see no further need to address this issue. Still, I would like to point out that a linear GLS model can handle AR1 errors, and would have been a viable choice since there is no interest in estimating the random effects variance anyway. Function gls() from R package nlme would offer a way to implement such a model.

With thanks to the reviewer for suggesting this approach, we have conducted a further sensitivity analysis using gls(). Since gls requires a nominal autocorrelation to be provided, we used a value of 0.01, which is a conservative estimate according to the ACF plot (see previous response to reviewers: estimated first order autocorrelation = 0.0047). With this putative first order autocorrelation, the p-value for the pandemic and policy are 0.04 and <0.001 for referrals; thus, as with other sensitivity analyses we have discussed, it becomes almost possible – by adjusting model parameters – to cast doubt on the association of the pandemic itself with the outcome, but hard to cast doubt on the association of the intervention (policy change) with the outcome.

We propose not to add this to the text because it uses a putative rather than estimated autocorrelation value.

4) In response to the original issue 6, the authors agree that ideally, rates should be based on the number of obese patients per GP per month, yet this information is not available. This limitation should be explicitly mentioned in the discussion.

We have added the following text:

> *Ideally we would calculate rates per 1000 people with obesity, but unfortunately this was not possible in this electronic health record database.*

5) In response to the original issue 7, the authors agree that GP practices that opted in to sharing of their data for the wholde time period may systematically differ from those practices not analyzed. This limitation sould be explicitly discussed as it is a question of how representative the obtained data are.

We have added the following remark to the limitations paragraph:

> *Similarly, the data does not identify which practices opted in to the incentive.*

6) In response to the original issue 7, the authors provide a reasonable explanation which is, however, speculative. As such, it should be mentioned in the limitations.

Done – see above.

7) In response to the original issue 11, the authors provide a reasonable argument. The issue remains, however, that individual SES data was not available, and this should be mentioned in the limitations.

We have changed

> *This analysis is subject to the usual limitations of routine health data.*

To

> *This analysis is subject to the usual limitations of routine health data, such as aggregate rather than individual-level information on socioeconomic status.*

8) p10 l187 "There was no evidence that the policy changed increased " -> delete "increased"

We have removed a spurious letter d so that it now reads:

> *There was no evidence that the policy change increased the use of weight management advice codes …*

REVIEWER #2

I have reviewed the responses to the reviewers' comments and I am satisfied that the comments have all been satisfactorily addressed.

I note the addition at line 156 "and in a log-scale model examining multiplicative effects (Supplement Table 1, column 3)." but do not see that this column has been included. Please correct.
Also note typo line 190: 'changed' should be 'change'.

Thank you – we chose not to include the extra column because of the change in units, but did not correct the cross-reference. We have removed the cross-reference to a non-existent column and inserted the p-values, so that the relevant sentence now reads:

> *This remained true in the alternative model that did not adjust for calendar month (Supplement Table 1, column 2) and in a log-scale model examining multiplicative effects (p<0.001 both for change at time of pandemic and for change at time of intervention).*
>
> We have corrected the typo in line 190 as noted above.

OTHER CHANGES

In this iteration we provide a new version of Figure 2 in which the axes are labelled by date, for consistency with Figure 1 and Figure 3.

VERSION 3 - REVIEW

**Reviewer**	**1**
**Name**	**Wollschlaeger, Daniel**
**Affiliation**	**Institute for Medical Biostatistics, Epidemiology and Infomatics, University Medical Center, Johannes Gutenberg University Mainz, Biostatistics**
**Date**	**18-Jan-2026**
**COI**	

I thank the authors for responding to my earlier comments. While the statistical analysis remains somewhat suboptimal, it appears unlikely that the overall conclusions critically depend on the particular modeling approach. Therefore, I support publication. Still, the authors should comment on potentially better modeling approaches in the discussion, thus enabling the readers to draw their own conclusions.

VERSION 3 - AUTHOR RESPONSE

REVIEWER #1

I thank the authors for responding to my earlier comments. While the statistical analysis remains somewhat suboptimal, it appears unlikely that the overall conclusions critically depend on the particular modeling approach. Therefore, I support publication. Still, the authors should comment on potentially better modeling approaches in the discussion, thus enabling the readers to draw their own conclusions.

We have added the following text to the limitations section of the Discussion (line 207-215):

"The model pre-specified in our analysis plan was consistent with our previous paper, but not necessarily optimal. Our sensitivity analyses explored some but not all potentially useful variations to the model (for example, generalised estimating equations). Given that the modelling details do not affect our graphs; that our p-values for the intervention are conclusively significant or conclusively non-significant ($p<0.001$ in both primary and secondary analyses); and that our interpretation does not rest on the coefficients, such changes would not affect our conclusions. The primary limitation remains that this study design can only show association but not prove causality."
